# The mediating roles of academic self-efficacy and learning interest in the relationship between teaching style and math behavior engagement among junior high school students in China

**DOI:** 10.1371/journal.pone.0311959

**Published:** 2024-10-14

**Authors:** Qing Geng, Mansour Amini, Siti Nur Aafifah Binti Hashim, Mengjun Zhu

**Affiliations:** 1 Faculty of Social Sciences & Liberal Arts, UCSI University, Kuala Lumpur, Malaysia; 2 School of Languages, Literacies and Translation, Universiti Sains Malaysia, Penang, Malaysia; National University of Lesotho, LESOTHO

## Abstract

Teacher guidance can positively impact students’ learning interest and mathematical behavior engagement. As a crucial part of classroom teaching, effective teaching styles play an indispensable role in middle school students’ mathematical behavior engagement. This study addresses the gap in understanding how different teaching styles influence junior high school students’ math behavior engagement in China, by examining the underexplored mediating roles of academic self-efficacy and learning interest in this relationship, which are critical yet often overlooked factors in fostering student engagement and improving educational outcomes in mathematics. Students from grades 7 to 9 in six middle schools in Jiangsu Province, China participated in the survey. The results indicate that: (1) academic self-efficacy mediates the relationship between effective teaching styles (humorous and lively style, rigorous and logical style, caring and sharing style) and mathematical behavior engagement among Chinese middle school students; (2) math learning interest mediates the relationship between effective teaching styles (humorous and lively style, rigorous and logical style, caring and sharing style, innovative and exploratory style) and mathematical behavior engagement among Chinese middle school students. Recommendations include encouraging teachers to adopt diverse teaching styles that foster both self-efficacy and interest.

## Introduction

Mathematics plays a crucial role in personal, educational, and societal advancement, with mathematical abilities being central to navigating today’s digital society [1]. Despite its importance, many students face cognitive and emotional challenges in mathematics, which can impede their learning and long-term success [2]. Various factors such as student motivation, teacher-student relationships, and family support significantly influence mathematics learning [3]. However, research indicates a declining trend in students’ motivation and engagement in mathematics during middle school [3], accompanied by decreasing performance [4,5]. Disengaged students often exhibit poor performance, boredom, and, in extreme cases, may even drop out. These issues underscore the importance of understanding the factors that can promote mathematics learning, particularly in middle school, when students’ engagement levels tend to wane [6].

Behavioral engagement has been identified as a critical factor influencing student learning outcomes [7], and educators must understand how to foster it effectively [8]. Students’ academic achievement is closely tied to their engagement in classroom activities, which enhances their understanding of course content [9]. Yet, disengagement remains a widespread issue among middle and high school students [10]. Given that engagement in mathematics learning is key to achieving long-term academic success in the subject, the role of teachers in fostering such engagement is pivotal [11]. Teachers’ use of appropriate teaching styles and strategies is a vital component of this process, shaping students’ learning experiences and success [12].

Research has shown that innovation in education and the cultivation of innovative talents are directly linked to teachers’ ability to adapt and personalize their teaching styles [13]. Effective teaching methods can foster classroom interaction, stimulate students’ interest in learning, and promote academic success [14]. Grasha’s widely recognized classification of teaching styles includes categories such as formal authority, expert, facilitator, personal model, and delegator [15]. In contrast, Chinese scholar He Wen classifies teaching styles into two broad categories—positive and efficient, or negative and inefficient—based on their contribution to student development. She further proposes four teaching styles: humorous and lively, rigorous and logical, caring and sharing, and innovative and exploratory [16].

These teaching styles impact students’ cognitive, emotional, and academic development in distinct ways. For example, a humorous and lively teaching style fosters a relaxed classroom environment, enhancing students’ academic self-efficacy [17]. Teachers’ care and emotional support have been shown to strengthen the teacher-student bond, which positively affects academic performance [18,19]. Furthermore, rigorous and logical teaching enhances students’ cognitive development, particularly in mathematics, where meticulous explanations are key to understanding complex concepts [20].

Despite the recognition of teaching styles as a critical factor in student engagement, research addressing how specific teaching styles impact middle school students’ engagement in mathematics is limited. This gap is significant, as understanding these relationships can provide insights into improving math education during a crucial developmental stage. This study, guided by Social Cognitive Theory (SCT) [21] and Self-Determination Theory (SDT) [22], seeks to fill this gap by examining the relationship between four teaching styles—humorous and lively, rigorous and logical, caring and sharing, and innovative and exploratory—and middle school students’ behavioral engagement in mathematics. It also explores whether academic self-efficacy and interest in mathematics mediate these relationships. By addressing this gap, the study contributes to the advancement of educational practices, offering educators evidence-based strategies to enhance student engagement and success in mathematics. Mathematics was chosen as the focus of this study due to its foundational role in both academic achievement and everyday life. Mathematics is a critical skill that cuts across disciplines, influencing performance in science, technology, engineering, and economics. Furthermore, mathematics consistently poses significant challenges for students by declining engagement and achievement during middle school. This period is particularly crucial as it sets the foundation for more advanced studies, and difficulties in math often lead to broader academic struggles. Given its importance and the widespread issues of disengagement, mathematics serves as an ideal subject to explore the influence of teaching styles on behavioral engagement. By focusing on mathematics, this study seeks to offer targeted insights that can be applied to a subject that is both a cornerstone of education and a common source of academic difficulties.

## Literature review

### Teacher teaching styles and student math engagement

Teaching style is defined as the consistent behavior of teachers in their interactions with students during the teaching process [23]. Research from various educational settings highlights that no single teaching method is universally effective across all subjects and student groups. Teachers generally exhibit a dominant or preferred teaching style but often blend elements from multiple styles to adapt to students’ diverse learning needs [24]. It has been widely recognized that students approach learning activities differently, and these differences necessitate flexibility in teaching methods to optimize learning outcomes [25]. Teachers should not rely solely on their preferred teaching methods but should understand that a single method may not meet the needs of all students [26]. Effective teaching styles have a positive impact on students’ academic performance, helping them to better understand and master knowledge [27]. When a teacher’s teaching style aligns with students’ learning styles, students’ understanding and memory improve, and they are more willing to engage in learning [28]. Teaching styles not only influence students’ academic performance but also affect their attitudes and engagement in learning. According to a study in Romania, students exhibit varying preferences for learning styles, and when teaching styles align with these preferences, it can lead to improvements in learning [29]. For example, a study in Pakistan found that students are more enthusiastic in class and have better performance when they think their teacher cares about their success [30]. Another study in Romania reported significant differences between students with auditory, visual and practical learning styles depending on the academic performance and the year of study [31].

Innovative teachers have unique insights into teaching, and novel teaching methods can attract students’ attention, making them more focused on lessons and thereby enhancing their behavioral engagement [32]. In biology classrooms, increased care and attention from teachers have been shown to make students feel valued, leading to higher grades and better engagement [33]. Additionally, when teachers are rigorous in their thinking, clear in their logic, and explicit in their teaching objectives, it helps students grasp key points and difficulties, leading to better academic performance [34].

Given the significant impact of teaching styles on student engagement and performance, this study explores the relationship between various teaching styles (humor-lively, rigorous-logical, caring-sharing, and innovative-exploratory) and students’ behavioral engagement in mathematics. Investigating this relationship is essential for adjusting teaching strategies to foster student participation, thereby improving educational outcomes and setting a foundation for future academic and career success. In diverse educational systems globally, effective teaching styles are recognized as critical external factors for enhancing student engagement in the classroom.

### The mediating effect of academic self-efficacy

Academic self-efficacy is defined as an individual’s belief and evaluation of their capability to accomplish learning tasks [21]. This study examines self-efficacy in mathematics, specifically referring to students’ belief in their capacity to successfully complete mathematical tasks and their academic performance [35]. Research has shown that in a more caring, challenging, and skill-oriented classroom environment, students’ math self-efficacy is significantly enhanced [36]. Students who possess higher levels of self-efficacy generally perform better academically [37,38]. Additionally, during the learning process, academic self-efficacy affects students’ choice of behavior, level of effort, and persistence when facing tasks, as well as their thinking and emotional responses when solving problems [39].

On one hand, academic self-efficacy directly influences students’ performance in math classes through activities such as active thinking, discussion, and proactively facing challenges. Students with stronger self-efficacy work harder, and when they encounter difficulties, they find ways to solve them, thereby promoting their engagement in classroom behavior. Conversely, students with lower self-efficacy, even if capable of completing a learning task, may doubt their abilities and easily give up, reducing their participation in classroom behavior [40]. On the other hand, academic self-efficacy influences students’ learning motivation and behavioral engagement in academic tasks [41]. Furthermore, academic self-efficacy has been found associated with various factors, such as classroom atmosphere, teaching practices, academic emotions, extracurricular tutoring, and students’ academic performance [42–44]. This mediating role suggests that fostering self-efficacy in students can amplify the positive effects of effective teaching on student engagement and achievement.

### The mediating effect of learning interest

Although research supports the predictive role of teacher style on learning engagement, the internal mechanisms of this influence remain limited. In this study, mathematical learning interest is defined as a cognitive inclination with emotional hues that individuals hold while striving to understand and explore mathematical knowledge during the learning process [41]. Student interest in learning is a crucial factor affecting their learning behavior and performance [45]. As the organizers, guides, and implementers of classroom teaching, teachers’ teaching abilities and professional knowledge directly influence students’ learning interest and effectiveness.

Research shows that if teachers select appropriate material difficulty and style based on students’ different learning styles, they can not only enhance students’ learning interest but also significantly improve their learning efficiency [46]. When teachers adopt more learner-centered teaching methods, learners’ interest in learning increases [47]. Humorous and witty teaching language can stimulate students’ initiative and enthusiasm for learning political knowledge, thereby sparking their interest in learning. When teachers support students’ autonomy, their learning interest also increases [48]. According to the self-determination theory, when students’ basic psychological needs are met, their motivation increases, thereby influencing their learning behavior. Thus, when learning interest as a motivational variable improves, students are more proactive in using their time for learning.

Research indicates that student learning interest is an important mediating variable in the relationship between a teacher’s teaching behavior and student academic performance [5,49]. Teachers’ teaching behavior can indirectly influence students’ academic performance by affecting student learning interest [50]. The junior high school years are crucial for the formation and development of students’ learning interest. The greater abstractness and strong logic of junior high school mathematics can easily lead to polarization in students’ mathematics learning. If teachers guide students properly, the students can easily develop a strong interest in learning mathematics. Conversely, they can easily lose interest in learning mathematics or even give up on it. Thus, this study aims to explore the mediating roles of academic self-efficacy and learning interest in the relationship between teaching styles and student engagement in mathematics. By drawing on literature from different regions, the study addresses gaps in existing research and contributes to a more generalized understanding of the factors influencing student engagement and performance.

**Fig 1:**
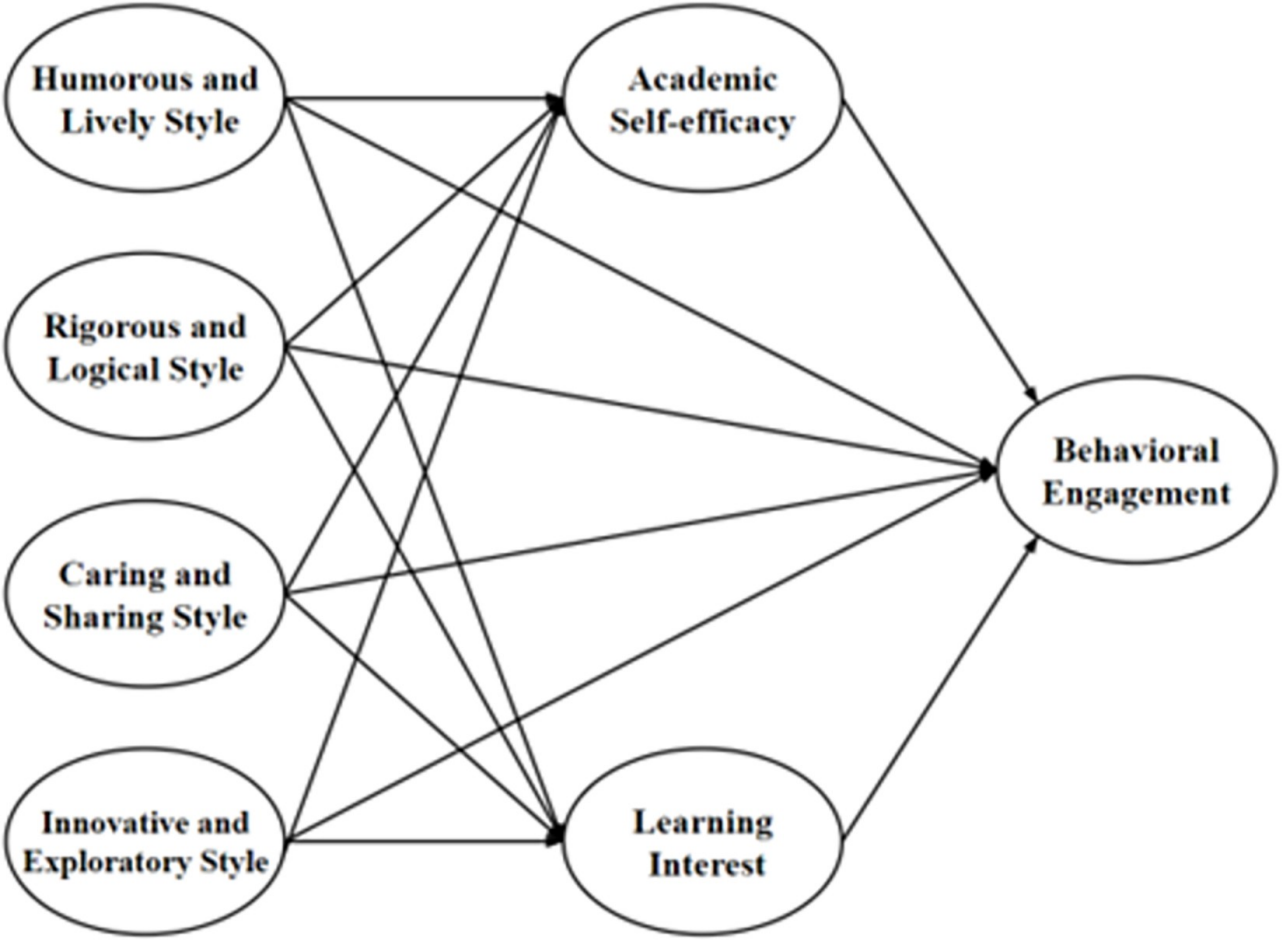
The conceptual model. In Fig 1 all arrows indicate hypothesized positive relationships.

According to SCT and SDT, environment, personal factors, and behavior interact. A teacher’s teaching style, as an environmental factor, can enhance students’ academic self-efficacy and learning interest, enabling students to focus on their studies. The hypothesized model is shown in Fig 1. The current study proposes the following hypotheses.

H1: The humorous and lively teaching style has a significant impact on mathematics behavioral engagement among junior high school students.

H2: The rigorous and logical teaching style has a significant impact on mathematics behavioral engagement among junior high school students.

H3: The caring and sharing teaching style has a significant impact on mathematics behavioral engagement among junior high school students.

H4: The innovative and exploratory teaching style has a significant impact on mathematics behavioral engagement among junior high school students.

H5: Academic self-efficacy mediates the relationship between humorous and lively teaching style and mathematics behavioral engagement.

H6: Academic self-efficacy mediates the relationship between rigorous and logical teaching style and mathematics behavioral engagement.

H7: Academic self-efficacy mediates the relationship between caring and sharing teaching style and mathematics behavioral engagement.

H8: Academic self-efficacy mediates the relationship between innovative and exploratory teaching style and mathematics behavioral engagement.

H9: Learning interest mediates the relationship between humorous and lively teaching style and mathematics behavioral engagement.

H10: Learning interest mediates the relationship between rigorous and logical teaching style and mathematics behavioral engagement.

H11: Learning interest mediates the relationship between caring and sharing teaching style and mathematics behavioral engagement.

H12: Learning interest mediates the relationship between innovative and exploratory teaching style and mathematics behavioral engagement.

## Method

### Data collection and sample

A stratified random sampling technique was employed to ensure a representative sample across different schools and grade levels. The population was divided into subgroups (strata) based on school location (urban, rural) and grade. Random samples were drawn from each stratum to reflect the diversity of the population. This method enhances the generalizability of the results and ensures the possibility of replicating the study in similar contexts. The participants were selected from junior high school students (grades 7 to 9) in China. Since students were between 13 to 16 years old, their parent/guardians were given written consent forms to sign and allow the students to participate in the study. Participation was voluntary and the form assured the study would not cause any risk or harm to the respondents. Likewise, it was specified on the consent form that the respondents’ answers would only be used for research purpose, anonymously with strict confidentiality and ethically. Confidentiality and anonymity were maintained throughout the research process, ensuring the protection of participants’ data. The ethics approval was obtained from Ethics Committee of Zhejiang Chinese Medical University on 5 April 2024. The data was collected from 22 April 2024 to 16 May 2024. A total of 1,025 valid samples were obtained in this study, including 595 males (58.0%) and 430 females (42.0%). There were 360 students in grade 7 (35.1%), 356 students in grade 8 (34.7%), and 309 students in grade 9 (30.1%) (S1 File).

Data was gathered using a structured questionnaire designed to measure teaching styles, academic self-efficacy, learning interest, and behavioral engagement. The questionnaire was validated and underwent a pilot test to ensure reliability and validity. SmartPLS 4 software was used for data analysis, following a two-stage process: evaluation of the measurement model and assessment of the structural model.

The measurement model was assessed for internal consistency, convergent validity, and discriminant validity using Cronbach’s alpha, composite reliability (CR), average variance extracted (AVE), and the Fornell-Larcker criterion. The structural model was evaluated using variance inflation factors (VIF) to assess collinearity and the Bootstrap method to test the significance of the paths. Furthermore, the study utilized the fsQCA approach to explore nonlinear relationships. This detailed explanation of the research design, sampling procedures, ethical considerations, and analytical tools provides a comprehensive understanding of the methodology, ensuring replicability and clarity in the presentation of the study.

While this study provides valuable insights into the relationship between teaching styles, self-efficacy, and behavioral engagement, the generalizability of the findings may be subject to certain limitations. The sample population is drawn from a specific educational context, which may not represent the diversity of student populations across different educational systems or cultural settings. As such, caution should be exercised when applying the findings to other settings or disciplines. Additionally, the use of PLS-SEM offers predictive validity within the model, but further studies across varied contexts and with different student groups are recommended to enhance the robustness and broader applicability of the results. Future research could also explore longitudinal data to examine how these relationships evolve over time, which would contribute to a more comprehensive understanding of the generalizability of the outcomes.

### Measures

To assess the effective teaching styles of teachers, this study used the scale developed by Him et al. (2011) [16] based on the characteristics of Chinese teachers. This scale comprises 18 measurement indicators: 6 items for the humorous and lively teaching style, 5 items for the rigorous and logical teaching style, 4 items for the caring and sharing teaching style, and 3 items for the innovative and exploratory teaching style.

The academic self-efficacy scale adapted from Liang (2000) [51] was used to evaluate the students’ self-efficacy in mathematics. This scale consists of 22 items. The mathematics learning interest scale, developed by Wu and Liu (2017) [52], consists of 17 items. The measurement of mathematical behavioral engagement adopted the learning engagement scale developed by Fredricks et al. (2016)[53], specifically using its behavioral engagement subscale, which consists of 11 items to assess students’ mathematical behavioral engagement. All scales were rated on a 5-point scale, i.e., 1 (strongly disagree) to 5 (strongly agree).

### Data analysis

This study employed Partial Least Squares Structural Equation Modeling (PLS-SEM). Due to its strong predictive ability, PLS-SEM is particularly suitable for complex models [54]. Therefore, this study used SPSS 26.0 and SmartPLS 4 for statistical analysis.

Given the comprehensive and complex nature of the factors influencing behavioral engagement, this study employed a non-linear research approach to explore the intricate relationships between the factors influencing behavioral engagement Therefore, this study further employed Fuzzy Set Qualitative Comparative Analysis (fsQCA) to analyze the complex relationships affecting behavioral engagement.

## Results

This study adopted a quantitative research approach, utilizing Partial Least Squares Structural Equation Modeling (PLS-SEM) to analyze the relationships between teaching styles, academic self-efficacy, learning interest, and behavioral engagement. The study followed a non-experimental, cross-sectional design, collecting data at a single point in time from a sample of junior high school students in China. To analyze the PLS path model, this study used SmartPLS 4 software. The results were interpreted in two stages: (1) evaluation of the measurement model, and (2) assessing of the structural model.

### Measurement model

This study assessed the reflective measurement model through internal consistency (Cronbach’s Alpha), composite reliability (CR), average convergent validity (AVE), and the outer loadings of the indicators. Discriminant validity was measured using the Fornell-Larcker criterion and the Heterotrait-Monotrait ratio (HTMT) [55]. The results showed that the Cronbach’s alpha coefficients of all measurement variables were higher than 0.7 (minimum value = 0.763), and the CR were all higher than 0.7 (minimum value = 0.831), indicating good internal consistency of the measurement model [56]. The outer loadings of all measurement model indicators were greater than 0.7 (minimum value = 0.704), and the AVE was greater than 0.5 (minimum value = 0.631), as shown in Table 1, demonstrating good convergent validity of the measurement model. The HTMT ratios between constructs did not exceed 0.85 (Table 2). Additionally, the square root values of the AVE for each variable were greater than the latent variable correlation coefficients between variables, indicating good discriminant validity among the variables in this study.

**Table 1:** Measurement model.

Construct	Outer Loading [min-max]	Cronbach’s alpha	CR	AVE
**First Order**				
Humorous and Lively Style	0.723–0.869	0.900	0.923	0.668
Rigorous and Logica Style	0.766–0.867	0.882	0.914	0.681
Caring and Sharing Style	0.773–0.857	0.837	0.891	0.672
Innovative and Exploratory Style	0.794–0.841	0.763	0.864	0.679
Self-efficacy in Learning Ability	0.711–0.846	0.944	0.952	0.642
Self-efficacy in Learning Behavior	0.732–0.856	0.947	0.954	0.653
Triggered Situational Interest	0.791–0.837	0.872	0.907	0.661
Maintained Situational Interest	0.704–0.844	0.856	0.897	0.637
Individual Interest	0.762–0.818	0.902	0.923	0.631
Behavioral Engagement	0.740–0.834	0.942	0.950	0.632
**Second Order**				
Academic Self-efficacy	0.841–0.846	0.944	0.831	0.711
Learning Interest	0.745–0.845	0.913	0.847	0.650

In Table 1, CR = composite reliability, and AVE = average variance extracted.

**Table 2:** Means, standard deviations, correlations, and discriminant validity results.

	Mean	SD	01	02	03	04	05	06	07	08	09	10
01 HLS	3.872	0.892	**0.818**	0.522	0.567	0.507	0.269	0.206	0.283	0.349	0.307	0.390
02 RLS	4.017	0.876	0.464	**0.825**	0.580	0.493	0.312	0.264	0.324	0.383	0.326	0.413
03 CSS	3.894	0.920	0.494	0.499	**0.820**	0.557	0.285	0.234	0.285	0.369	0.318	0.440
04 IES	3.820	0.940	0.420	0.404	0.446	**0.824**	0.242	0.200	0.362	0.400	0.345	0.414
05 SLA	3.317	0.942	0.248	0.286	0.254	0.205	**0.801**	0.445	0.306	0.278	0.284	0.354
06 SLB	3.153	0.910	0.191	0.242	0.209	0.169	0.423	**0.808**	0.244	0.264	0.288	0.400
07 TSI	3.969	0.906	0.253	0.285	0.247	0.295	0.277	0.222	**0.813**	0.578	0.453	0.373
08 MSI	2.574	0.934	-0.309	-0.332	-0.314	-0.324	-0.251	-0.238	-0.502	**0.798**	0.606	0.476
09 II	2.699	0.934	0.278	0.291	0.277	0.287	0.262	0.266	0.402	-0.534	**0.794**	0.399
10 BE	3.449	0.911	0.361	0.378	0.391	0.351	0.335	0.378	0.339	-0.429	0.368	**0.795**

In Table 2, the diagonal in bold represents the square root of the average variance extracted (AVE). Below the diagonal elements are the correlations between the construct values. Above the diagonal elements are the HTMT values. HLS = Humorous and Lively Style; RLS = Rigorous Logical Style; CSS = Caring and Sharing Style; IES = Innovative Exploratory Style; SLA = Self-efficacy in Learning Ability; SLB = Self-efficacy in Learning Behavior; TSI = Triggered Situational Interest; MSI = Maintained Situational Interest; II = Individual Interest; BE = Behavioral Engagement.

### Structural model

The analysis of the structural model was based on the methods of Hair et al. [54]. The analysis showed that the variance inflation factor (VIF) for each predictor variable in the model had a maximum value of 1.616, which is less than 5 (Table 3), indicating no significant collinearity among the endogenous variables.

**Table 3:** Hypothesis test results.

Hypothesis		Bootstrap β	S.E.	t	P	95%CI		f^2^	VIF	Results
						Lower	Upper			
**BE: R**^**2**^ **= 0.352 AdjR**^**2**^ **= 0.348 Q**^**2**^ **= 0.220**										
H1	HLS→BE	0.080	0.034	2.346	0.019	0.014	0.147	0.007	1.530	Supported
H2	RLS→BE	0.078	0.034	2.307	0.021	0.010	0.142	0.006	1.565	Supported
H3	CSS→BE	0.124	0.032	3.942	<0.001	0.065	0.188	0.015	1.616	Supported
H4	IES→BE	0.086	0.032	2.705	0.007	0.024	0.151	0.008	1.428	Supported
	ASE→BE	0.232	0.028	8.397	<0.001	0.178	0.286	0.068	1.224	
	LI→BE	0.249	0.030	8.395	<0.001	0.191	0.308	0.070	1.372	
**ASE: R**^**2**^ **= 0.127 AdjR**^**2**^ **= 0.123 Q**^**2**^ **= 0.054**										
	HLS→ASE	0.094	0.040	2.374	0.018	0.018	0.173	0.007	1.505	
	RLS→ASE	0.193	0.035	5.453	<0.001	0.125	0.263	0.029	1.497	
	CSS→ASE	0.107	0.039	2.766	0.006	0.030	0.183	0.008	1.594	
	IES→ASE	0.057	0.035	1.634	0.102	-0.009	0.127	0.003	1.377	
**LI: R**^**2**^ **= 0.221 AdjR**^**2**^ **= 0.218 Q**^**2**^ **= 0.092**										
	HLS→LI	0.128	0.037	3.414	0.001	0.056	0.202	0.014	1.505	
	RLS→LI	0.182	0.038	4.757	<0.001	0.106	0.259	0.028	1.497	
	CSS→LI	0.103	0.036	2.859	0.004	0.033	0.174	0.009	1.594	
	IES→LI	0.198	0.036	5.497	<0.001	0.129	0.269	0.037	1.377	

Using the Bootstrap method with 5000 resamples, we analyzed the significance test results for the paths (Table 2). Humorous and lively, rigorous and logical, caring and sharing, innovative and exploratory teaching styles, academic self-efficacy, and learning interest all had a significant positive impact on behavioral engagement (P<0.05), with standardized coefficients of 0.008, 0.078, 0.124, and 0.086, respectively, confirming hypotheses H1, H2, H3, and H4. Additionally, academic self-efficacy and learning interest had a significant positive impact on behavioral engagement (P<0.001).

Humorous and lively, rigorous and logical, and caring and sharing teaching styles had a significant positive impact on academic self-efficacy (P<0.05). However, the innovative and exploratory teaching style did not have a significant impact on academic self-efficacy (P>0.05). Humorous and lively, rigorous and logical, caring and sharing, and innovative and exploratory teaching styles, along with academic self-efficacy and learning interest, had a significant positive impact on learning interest (P<0.01).

### Mediating role of academic self-efficacy and learning interest

This study employed the coefficient product method to test for mediation effects. This method has higher testing power compared to the stepwise testing method and the coefficient difference method [57]. Meanwhile, we used the bias-corrected Bootstrap method (Percentile Bootstrap Method) in this study to estimate the 95% confidence interval (CI) for the mediation effect obtained by the coefficient product method. If the 95% CI does not include 0, the effect is considered significant [58].

The analysis results showed the mediating effect of academic self-efficacy between humorous and lively teaching style and behavioral engagement, 95% CI = [0.004, 0.042]. Since the confidence interval does not include 0, it indicates a significant mediating effect of academic self-efficacy between the humorous and lively teaching style and behavioral engagement, with a standardized mediating effect of 0.022 (Table 4), supporting hypothesis H5.

**Table 4:** Mediation effect test.

Direct Path		Bootstrap β	S.E.	95%CI		Hypothesis	Results
				Lower	Upper		
HLS→BE	Total Effect	0.134	0.038	0.060	0.210		
	Direct Effect	0.080	0.034	0.014	0.147		
	Total Indirect Effect	0.054	0.016	0.024	0.086		
	HLS→ASE→BE	0.022	0.010	0.004	0.042	H5	Supported
	HLS→LI→BE	0.032	0.010	0.013	0.053	H9	Supported
RLS→BE	Total Effect	0.168	0.035	0.099	0.236		
	Direct Effect	0.078	0.034	0.010	0.142		
	Total Indirect Effect	0.090	0.016	0.061	0.123		
	RLS→ASE→BE	0.045	0.010	0.027	0.066	H6	Supported
	RLS→LI→BE	0.045	0.011	0.025	0.068	H10	Supported
CSS→BE	Total Effect	0.175	0.036	0.105	0.248		
	Direct Effect	0.124	0.032	0.065	0.188		
	Total Indirect Effect	0.050	0.014	0.023	0.079		
	CSS→ASE→BE	0.025	0.010	0.007	0.044	H7	Supported
	CSS→LI→BE	0.026	0.010	0.008	0.045	H11	Supported
IES→BE	Total Effect	0.149	0.035	0.082	0.218		
	Direct Effect	0.086	0.032	0.024	0.151		
	Total Indirect Effect	0.063	0.015	0.035	0.094		
	IES→ASE→BE	0.013	0.008	-0.002	0.030	H8	Not supported
	IES→LI→BE	0.049	0.011	0.030	0.072	H12	

Similarly, academic self-efficacy mediates the relationship between rigorous and logical teaching style and behavioral engagement (95% CI = [0.027, 0.066], β = 0.045), supporting hypothesis H6; it also mediates the relationship between caring and sharing teaching style and behavioral engagement (95% CI = [0.007, 0.044], β = 0.045), supporting hypothesis H7. However, academic self-efficacy does not mediate the relationship between innovative and exploratory teaching style and behavioral engagement (95% CI = [-0.002, 0.030]), as the confidence interval includes 0, so hypothesis H8 is not supported.

Furthermore, learning interest mediates the relationship between humorous and lively teaching style and behavioral engagement (95% CI = [0.013, 0.053], β = 0.032), supporting hypothesis H9; it also mediates the relationship between rigorous and logical teaching style and behavioral engagement (95% CI = [0.025, 0.068], β = 0.045), supporting hypothesis H10; and it mediates the relationship between caring and sharing teaching style and behavioral engagement (95% CI = [0.008, 0.045], β = 0.026), supporting hypothesis H11. Finally, learning interest mediates the relationship between innovative and exploratory teaching style and behavioral engagement (95% CI = [0.030, 0.072], β = 0.049), supporting hypothesis H12.

### Predictive validity of PLS path model

**Fig 2:**
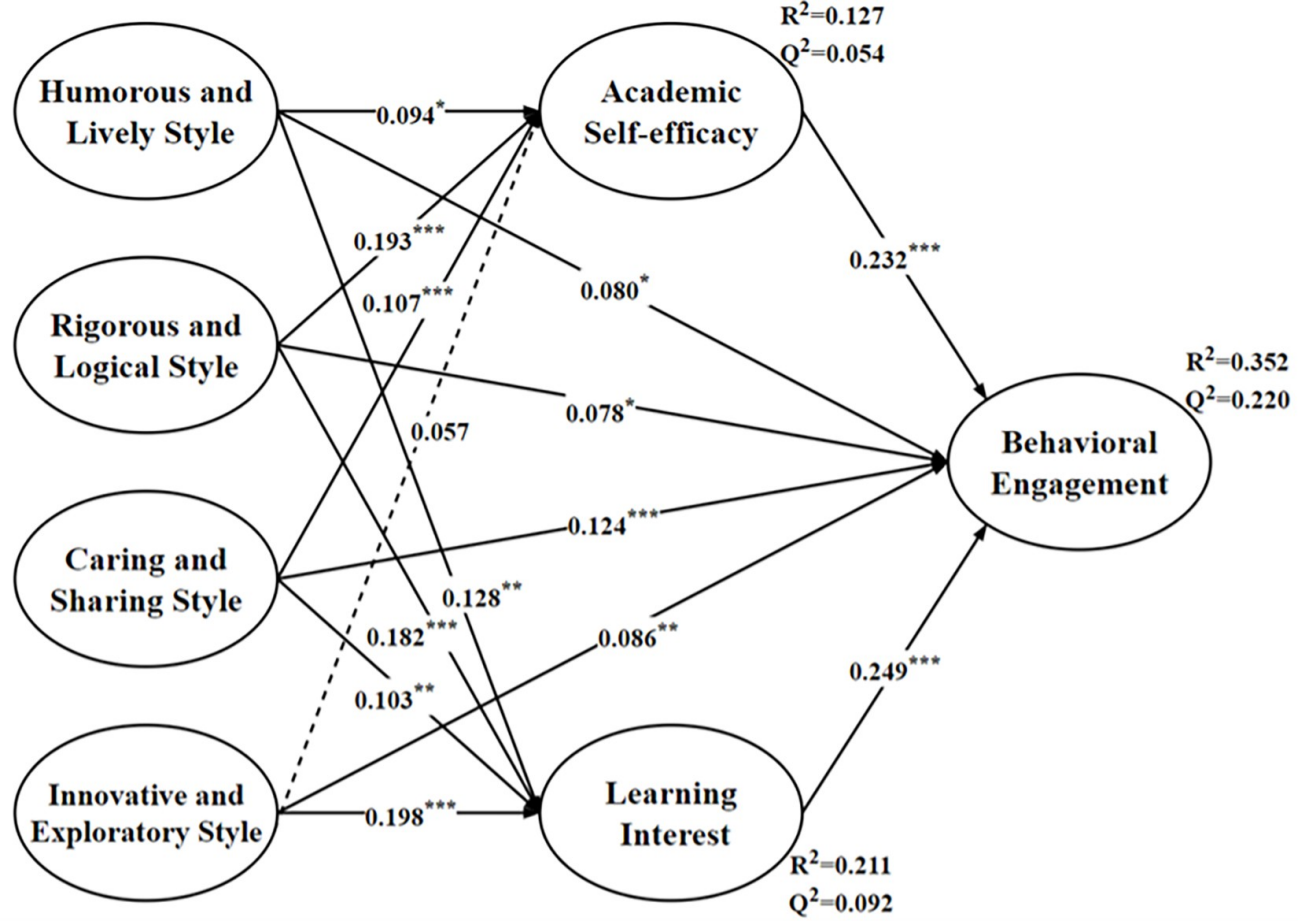
Structural equation model. In Fig 2, * indicates P<0.05, ** indicates P<0.01, *** indicates P<0.001, and dashed lines indicate non-significance.

In the research model (Fig 2), the R^2^ values for academic self-efficacy, learning interest, and behavioral engagement are 0.127, 0.221, and 0.352, respectively. The Stone-Geisser Q^2^ values for academic self-efficacy, learning interest, and behavioral engagement are 0.057, 0.092, and 0.220, respectively. Since all Q^2^ values are greater than 0, this indicates that the model has good predictive relevance for the endogenous constructs.

In the evaluation of PLS predictive ability, the Q^2^ Predict values of the latent variables were first assessed. The Q^2^ Predict values for academic self-efficacy, learning interest, and behavioral engagement are 0.117, 0.212, and 0.224, respectively, all of which are greater than 0. This further confirms the predictive ability of the model. Secondly, the Q^2^ Predict values for each measurement indicator in the PLS-SEM model range from 0.028 to 0.169, all of which are greater than 0, indicating that each measurement indicator has predictive relevance. Additionally, in this study, the RMSE (Root Mean Square Error) and MAE (Mean Absolute Error) values of most measurement indicators in the PLS-SEM model are lower than those of the LM model, indicating that the model in this study has a medium predictive power [54].

### fsQCA approach

**Fig 3:**
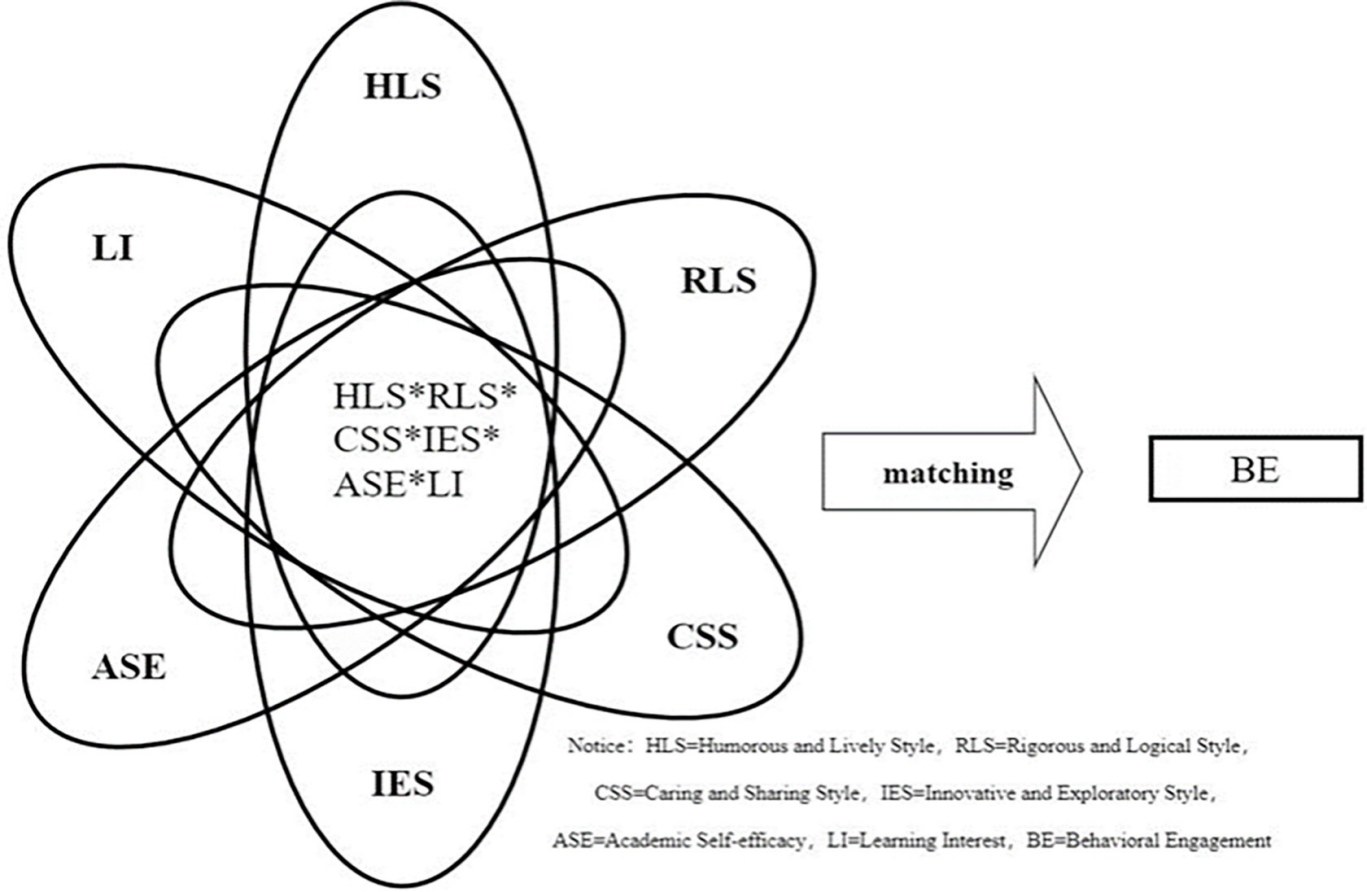
Nonlinear influence model of behavior engagement.

Given the comprehensive and complex nature of the factors influencing behavioral engagement, this study further employs fsQCA to analyze the intricate relationships affecting behavioral engagement (Fig 3). fsQCA uses Boolean algebra to generate causal condition combinations that lead to outcomes. The core of the fsQCA method is the calibration procedure and the truth table analysis. Calibration is the process of transforming conventional measures into fuzzy sets [59].

### Data calibration

This study used fuzzy set calibration for both condition and outcome variables. The direct calibration method was employed in this study, using the 95-50-5 calibration method, where the 95th percentile, median, and 5th percentile were set as the calibration anchors for full membership, crossover point, and full non-membership, respectively. Additionally, to prevent cases from being excluded due to a membership score of exactly 0.50 in any condition or outcome set, a constant of 0.001 was added to all conditions and outcomes with a membership score of 0.50 [60].

### Configuration analysis of high behavioral engagement

In the sufficiency analysis for configurations leading to high behavioral engagement as the outcome variable, the case frequency threshold was set to 10, the raw consistency threshold was set to 0.8, and the PRI consistency threshold was set to 0.65. As shown in Table 5, three configurations that can lead to high behavioral engagement were identified, consisting of a total of five sub-configurations. The overall consistency for generating high behavioral engagement in this study is 0.868, and the overall coverage is 0.616. The consistency levels of each configuration are above 0.8, indicating a high level of explanatory power for each configuration, meeting the research requirements. Following the process of configuration theorization [61], the configurations were named based on the core conditions included in each configuration: Universal Learning-Interest Type (S1), Humorous Caring Motivational Type (S2), and Rigorous Innovation Motivational Type (S3).

**Table 5:** Results from fsQCA.

	High behavioral engagement					Non-high behavioral engagement				
	S1	S2		S3		NS1	NS2	NS3	NS4	
		S2a	S2b	S3a	S3b				NS4a	NS4b
Humorous and Lively Style	●	●	●	*			x	#	#	#
Rigorous and Logical Style	●	*		●	●	#		#	#	#
Caring and Sharing Style	●	●	●		*	#	#	#		x
Innovative and Exploratory Style	●		*	●	●	#	x		#	#
Academic Self-efficacy		●	●	●	●	#	#	#	#	#
Learning Interest	●	●	●	●	●	#	#	#	x	
Raw coverage	0.500	0.479	0.465	0.471	0.473	0.358	0.372	0.358	0.355	0.350
Unique coverage	0.057	0.036	0.022	0.028	0.030	0.034	0.048	0.034	0.031	0.026
Consistency	0.891	0.900	0.902	0.903	0.899	0.918	0.913	0.923	0.916	0.909
Overall solution coverage	0.616					0.497				
Overall solution consistency	0.868					0.887				

In Table 5. ●indicates the presence of a core condition, * indicates the presence of a peripheral condition, # indicates the absence of a core condition, x indicates the absence of a peripheral condition, High behavioral engagement (case threshold = 10; raw = 0.80, PRI = 0.65), Non-high behavioral engagement (case threshold = 10; raw = 0.80, PRI = 0.65).

Configuration S1: Universal Learning-Interest Driven Type. Configuration S1 identifies the humorous and lively style, rigorous logical style, caring and sharing style, innovative exploratory style, and learning interest as core conditions. This configuration implies that regardless of the level of academic self-efficacy, high levels of humorous and lively style, rigorous logical style, caring, and sharing style, innovative exploratory style, and learning interest can lead to high behavioral engagement. This partially supports hypotheses H1-H4 and H9-H12.

Configuration S2: Humorous Caring Motivational Driven Type. Configuration S2a shows that high levels of humorous and lively style, caring and sharing style, academic self-efficacy, and learning interest are core conditions, with rigorous logical style as a peripheral condition, that can lead to high behavioral engagement. Without distinguishing between core and peripheral conditions, S2a is consistent with NS3 in non-high behavioral engagement. This partially supports hypotheses H1-H3, H5-H7, and H9-H11. Configuration S2b shows that high levels of humorous and lively style, caring and sharing style, academic self-efficacy, and learning interest are core conditions, with innovative exploratory style as a peripheral condition, that can lead to high behavioral engagement. Without distinguishing between core and peripheral conditions, the result can be validated with NS2 in non-high behavioral engagement. This partially supports all hypotheses except H2, H6, and H10.

Configuration S3: Rigorous Innovation Motivational Driven Type. Configuration S3a shows that high levels of rigorous logical style, innovative exploratory style, academic self-efficacy, and learning interest as core conditions, with humorous and lively style as a peripheral condition, can lead to high behavioral engagement. Without distinguishing between core and peripheral conditions, the result can be validated with NS4a in non-high behavioral engagement. This partially supports all hypotheses except H3, H7, and H11. Configuration S3b shows that high levels of rigorous logical style, innovative exploratory style, academic self-efficacy, learning interest, and caring and sharing style can lead to higher behavioral engagement. Without distinguishing between core and peripheral conditions, the result can be validated with NS1 in non-high behavioral engagement. This partially supports all hypotheses except H1, H5, and H9.

### Configuration analysis of non-high behavioral engagement

In the sufficiency analysis for configurations leading to non-high behavioral engagement, the case frequency threshold was set to 10, the raw consistency threshold was set to 0.8, and the PRI consistency threshold was set to 0.65. As shown in Table 5, five sub-configurations that can lead to non-high behavioral engagement were identified. The overall consistency for generating non-high behavioral engagement is 0.887, and the overall coverage is 0.497.

Except for NS4b, the non-high behavioral engagement configurations have been previously mentioned. Configuration NS4b indicates that under the absence of a humorous and lively style, rigorous logical style, innovative exploratory style, academic self-efficacy as core missing conditions, and caring and sharing style as a peripheral missing condition, behavioral engagement will not be high.

### Summary of analysis

This study provides insights valuable to both academicians and practitioners by offering empirical evidence on how different teaching styles, academic self-efficacy, and learning interest contribute to enhancing student behavioral engagement. These findings can guide educators in tailoring their teaching strategies to maximize student participation, motivation, and overall engagement, which are critical for academic success and retention.

To analyze the PLS path model, this study used SmartPLS 4 software. The results were interpreted in two stages: (1) evaluation of the measurement model, and (2) assessment of the structural model. This study assessed the reflective measurement model through internal consistency (Cronbach’s Alpha), composite reliability (CR), average convergent validity (AVE), and the outer loadings of the indicators. Discriminant validity was measured using the Fornell-Larcker criterion and the Heterotrait-Monotrait ratio (HTMT). The results confirmed the robustness of the measurement model, indicating that the teaching styles evaluated in this study are reliable predictors of student engagement, which is beneficial for academicians designing curricula and interventions. The analysis confirmed that humorous, rigorous, caring, and innovative teaching styles positively influence both academic self-efficacy and learning interest. These findings have direct practical applications for educators. For instance, teachers can adopt a more humorous or caring approach to foster a positive learning environment, thereby enhancing students’ confidence in their learning abilities. Practitioners in education policy can use these insights to advocate for professional development programs that encourage varied teaching styles tailored to students’ emotional and cognitive needs.

Furthermore, The model’s predictive validity, demonstrated by significant R^2^ and Q^2^ values, offers a reliable framework for educators and academic researchers interested in predicting student engagement outcomes based on teaching styles and self-efficacy. This model can inform academic interventions aimed at enhancing student motivation and persistence, thus benefiting both academicians developing engagement theories and practitioners implementing them in educational settings.

Several steps were taken to mitigate these generalizability concerns by employing Partial Least Squares Structural Equation Modeling (PLS-SEM) and Fuzzy Set Qualitative Comparative Analysis (fsQCA), which allowed us to capture complex, non-linear relationships between variables. While the local context does play a role, the theoretical framework underpinning the relationships between teaching styles, student engagement, and motivational factors is grounded in broader educational theories that may have applicability across different populations.

By making the findings applicable to real-world teaching scenarios, this research contributes to both the academic body of knowledge and the practical challenges educators face in diverse classroom environments.

## Discussion

### Direct relationships

This study sheds light on the impact of different teaching styles (humorous and lively, rigorous and logical, caring and sharing, and innovative and exploratory) on Chinese junior high school students’ behavioral engagement in mathematics. While previous studies have acknowledged the role of teaching styles in influencing student engagement, this research adds depth by differentiating between specific styles and examining their direct relationships with behavioral engagement in a cultural context unique to Chinese junior high school education. The findings confirm that all four teaching styles significantly influence students’ behavioral engagement, validating hypotheses H1 through H4 and aligning with existing studies in the broader educational literature [62]. However, the novelty of this study lies in the nuanced exploration of how each teaching style directly contributes to engagement, offering new insights that extend beyond traditional Western contexts. According to social cognitive theory [21], external factors such as teaching styles are pivotal in shaping students’ behavior. Teachers play an essential role in motivating and engaging students [63], and this study provides empirical evidence on how varied teaching approaches can differentially stimulate students’ behavioral engagement. For example, humorous and lively teaching encourages curiosity and active participation by creating a relaxed and engaging atmosphere [64], while teachers who use active teaching methods in the classroom employ diverse materials and encourage teamwork, leading to better student performance [65]. Moreover, rigorous and logical teaching enhances analytical thinking and problem-solving skills. When students feel the care of their teachers, they tend to be more autonomous, cooperative, and engaged in learning [66]. This differentiation underscores the importance of matching teaching methods to student needs in order to foster engagement, which, although previously noted, has not been explored in such detail within the Chinese educational context. By adopting new methods, students will be more focused and better able to retain information [67]. Moreover, this study not only confirms existing theoretical assertions but contributes to the body of knowledge by applying these principles in a new cultural and educational setting, thus highlighting the potential variability in teaching effectiveness across different cultural landscapes. High levels of academic self-efficacy make students more likely to maintain positive and sustained efforts when facing learning challenges. This belief is a key motivator for students to persist and ultimately succeed [68]. Likewise, positive interactions between teachers and students are crucial for improving students’ self-efficacy [69]. Such insights suggest that while the underlying theory may be universally applicable, the specific manifestations of teaching styles may vary depending on the demographic and cultural context.

### Mediating role of academic self-efficacy and learning interest

The mediating role of academic self-efficacy in the relationship between teaching styles (humorous and lively, rigorous and logical, caring and sharing) and behavioral engagement (Hypotheses 5, 6, and 7) is another novel contribution. The research not only aligns with social cognitive theory’s emphasis on self-efficacy [21] but extends previous findings by emphasizing that certain teaching styles can significantly enhance students’ confidence in their mathematical abilities. This provides new evidence for the interplay between teaching methods and academic self-efficacy, particularly in mathematics education—a subject where student confidence is often a critical factor in engagement. Interestingly, the study found that academic self-efficacy did not significantly mediate the relationship between innovative and exploratory teaching styles and behavioral engagement, likely due to the limited presence of such teaching styles in the observed classrooms. This finding highlights a potential area for future research and pedagogical reform, as the integration of innovative teaching methods remains underutilized in the Chinese context. Furthermore, the complementary fsQCA analysis, which identified innovative exploratory teaching and self-efficacy as core conditions affecting engagement, illustrates the value of mixed-method approaches in educational research, providing a more nuanced understanding of the data. Learning interest was also found to mediate the relationship between various teaching styles and behavioral engagement, supporting Hypotheses 9, 10, 11, and 12. Self-determination theory asserts that students’ motivational behaviors are shaped by their social environment [70], and this study confirms that teacher-student interactions significantly impact students’ learning interests. Importantly, this research highlights how different teaching styles can foster intrinsic motivation and transform students into active learners, providing new insights into how teaching methods can be optimized to enhance student interest in subjects like mathematics [71].

### Contribution to theoretical frameworks

By situating the findings within both social cognitive theory and self-determination theory, this study demonstrates how teaching styles can serve as key external factors influencing both self-efficacy and interest in learning. These two frameworks provide a robust theoretical foundation for understanding how teaching influences engagement, offering practical implications for educators. More importantly, this study enriches the application of these theories in a specific cultural and disciplinary context, Offering new perspectives on their utility beyond Western classrooms. Additionally, the research illustrates how different dimensions of teaching style can align with these theoretical constructs to drive student engagement, a contribution that deepens the theoretical discourse in educational psychology.

### Generalizability across academic disciplines

The findings of this study have broader implications that extend beyond mathematics education and can be replicated across other academic disciplines. For instance, teaching styles that promote engagement in mathematics could similarly enhance engagement in subjects like science, technology, and engineering, where problem-solving and logical thinking are equally crucial. The mediating roles of self-efficacy and learning interest also suggest that interventions aimed at improving these factors could enhance student engagement in a variety of subjects. Future research could explore how these findings apply in other disciplines to better understand the universal versus subject-specific effects of teach However, while the study offers valuable insights, issues of replication and generalizability should be considered. The sample was limited to Chinese junior high school students, which may restrict the generalizability of the findings to other contexts. Nonetheless, the study addresses this limitation by emphasizing the importance of cultural context in shaping the effectiveness of teaching styles. Future studies could replicate this research in different demographic and cultural settings to determine whether similar patterns of engagement emerging styles.

### Practical recommendations

For educators and practitioners, this study underscores the importance of adopting diverse and flexible teaching styles that cater to students’ varying needs. Teachers should incorporate humor, logical rigor, caring relationships, and innovation to create a learning environment that not only engages students but also supports their self-efficacy and interest in learning. Furthermore, professional development programs should emphasize the need for teachers to develop a range of teaching methods that can be adapted to different classroom contexts and student demographics. These insights provide a roadmap for educational practitioners to enhance student engagement, particularly in subjects that students often find challenging, such as mathematics. By tailoring teaching styles to the specific needs of students, educators can foster a more engaging and supportive learning environment that promotes both academic success and personal growth.

## Conclusions

This study represents a pioneering effort to investigate the impact of different teaching styles on academic self-efficacy, learning interest, and behavioral engagement in mathematics. Through a detailed analysis using structural equation modeling (SEM) and fuzzy set Qualitative Comparative Analysis (fsQCA), this research reveals that all four identified teaching styles significantly influence behavioral engagement in mathematics. Notably, while the relationship between the innovative-exploratory teaching style and academic self-efficacy was found to be insignificant in the SEM analysis, fsQCA demonstrated that this teaching style, combined with academic self-efficacy, serves as a core condition for enhancing students’ behavioral engagement. The application of fsQCA has provided novel insights into the configurations of teaching styles, academic self-efficacy, and learning interest that lead to high engagement levels, thus making a substantial theoretical contribution to the field.

This study introduces a new framework for understanding the interplay between teaching styles, academic self-efficacy, and learning interest, offering a comprehensive view of how these factors collectively influence students’ engagement in mathematics. The use of fsQCA to identify configurations that lead to high engagement adds depth to our theoretical understanding and highlights the importance of considering multiple interacting factors rather than isolated variables.

One limitation of this study is its cross-sectional design, which restricts the ability to draw causal inferences about the relationships between variables over time. Future research should employ longitudinal methodologies to explore how these relationships evolve and to better understand the causality between teaching styles, academic self-efficacy, and behavioral engagement. Additionally, expanding the study to include diverse educational contexts and populations could enhance the generalizability of the findings.

This research contributes to the field by providing a fresh perspective on how teaching styles and motivational factors interact to affect students’ engagement in mathematics. The identification of specific teaching style configurations that lead to increased engagement offers actionable insights for educators and contributes to the development of more effective teaching strategies.

To maximize the impact of their teaching, educators should consider adopting a range of teaching styles tailored to their students’ needs and characteristics. Emphasizing multidimensional approaches—such as rigor, care, humor, and innovation—can foster higher levels of self-efficacy and interest in learning. Additionally, ongoing professional development and support for teachers are crucial for helping them implement these strategies effectively. By integrating these recommendations into their practices, educators can enhance students’ academic engagement and overall learning experience.

This study strongly encourages the academic community to focus on engaged, practical scholarship that bridges the gap between theoretical insights and actionable teaching practices. By prioritizing research that addresses real-world educational challenges and offers practical solutions, scholars can contribute to the development of more effective and impactful teaching strategies.

## Supporting information

S1 File Data. (XLSX)
